# Effects of sodium fluoride on blood cellular and humoral immunity in mice

**DOI:** 10.18632/oncotarget.20198

**Published:** 2017-08-10

**Authors:** Hongrui Guo, Ping Kuang, Qin Luo, Hengmin Cui, Huidan Deng, Huan Liu, Yujiao Lu, Jing Fang, Zhicai Zuo, Junliang Deng, Yinglun Li, Xun Wang, Ling Zhao

**Affiliations:** ^1^ College of Veterinary Medicine, Sichuan Agricultural University, Ya’an 625014, China; ^2^ Key Laboratory of Animal Diseases and Environmental Hazards of Sichuan Province, Sichuan Agriculture University, Ya’an 625014, China

**Keywords:** NaF, blood, cellular immunity, humoral immunity, mice

## Abstract

Exposure to high fluorine can cause toxicity in human and animals. Currently, there are no systematic studies on effects of high fluorine on blood cellular immunity and humoral immunity in mice. We evaluated the alterations of blood cellular immunity and humoral immunity in mice by using flow cytometry and ELISA. In the cellular immunity, we found that sodium fluoride (NaF) in excess of 12 mg/Kg resulted in a significant decrease in the percentages of CD3^+^, CD3^+^CD4^+^, CD3^+^CD8^+^ T lymphocytes in the peripheral blood. Meanwhile, serum T helper type 1 (Th1) cytokines including interleukin (IL)-2, interferon (IFN)-γ, tumor necrosis factor (TNF), and Th2 cytokines including IL-4, IL-6, IL-10, and Th17 cytokine (IL-17A) contents were decreased. In the humoral immunity, NaF reduced the peripheral blood percentages of CD19^+^ B lymphocytes and serum immunoglobulin A (IgA), immunoglobulin G (IgG) and immunoglobulin M (IgM). The above results show that NaF can reduce blood cellular and humoral immune function in mice, providing an excellent animal model for clinical studies on immunotoxicity-related fluorosis.

## INTRODUCTION

Fluorine is necessary for bone health and dental caries at low doses in mammals and humans [1]. Fluorine is widely used as a cofactor in medicine, e.g., anesthetics, antibiotics, anti-cancer and anti-inflammatory agents, and psychopharmaceuticals [1, 2]. Fluorine is ubiquitous in food and water in varying amounts, and exposure to fluoride is a common phenomenon because it is a pollutant from industrial operations [3]. Fluorosis is a severe disease caused by fluoride toxicity that occurs in many areas [4]. It has been reported that millions of people are affected by fluoride toxicity, particularly in China. Exposure to high fluoride commonly occurs by ingestion of contaminated water [5]. The daily average fluorine intake by adults from food and water is estimated to be 1 mg if they live in a community with <0.7 ppm in the water, and about 2.7 mg if the water is fluoridated. The average dietary intake (including water) of fluoride ranges between 1.4 and 3.4 mg/day (0.02-0.048 mg/kg/day) for adults living in areas with 1.0 mg/L fluoride in the water. In areas with <0.3 mg/L fluoride in water, the adult dietary intake ranges from 0.3 to 1.0 mg/day (0.004-0.014 mg/kg/day). In children, the dietary intake ranges from 0.03 to 0.06 mg/kg/day in areas with fluoridated water and from 0.01 to 0.04 mg/kg/day in areas without fluoridated water [6].

Excessive fluoride intake may induce multiple organ and tissue damage including skeletal, nervous, digestive, respiratory, genitourinary and endocrine systems [7]. Fluoride ions can bind to functional amino acid groups surrounding the active center of an enzyme to cause an inhibitory effect, as is the case for enzymes of the glycolytic pathway and the Krebs cycle, which are sensitive to inhibition by fluoride [8]. Therefore, fluoride ions inhibit cellular respiration and decrease the production of ATP. Na/K-ATPases are also inhibited, leading to ATP depletion and a disturbance in cell membrane potential [8]. The effects of fluoride on glucose metabolism have been examined both *in vivo* and *in vitro* studies. It has been shown that fluoride overexposure may contribute to impaired glucose tolerance or increased blood glucose [9].

Our previous studies documented fluoride-induced oxidative damage and pathological injury in liver [10, 11], kidney [12], and intestine [13-16] in broiler chickens. Fluoride can induce oxidative damage, apoptosis and DNA damage in human osteosarcoma cells and in rat sertoli cells [17, 18]. In recent years, more studies have focused on the effect of fluorine on the immune system. Numerous studies have suggested that fluorine can induce functional impairment of immune system in human and animals [19-23]. High fluorine may adversely affect the immune organs including thymus [24, 25], spleen [25-27], bursa of Fabricius [28], cecal tonsil [29, 30] by reducing lymphocyte number, causing structural damage, inducing oxidative stress and apoptosis. Wang et al. [31] reported that excessive fluoride can induce apoptosis in the thymus of rats and inhibit immune function. Das et al. [20] demonstrated that sodium fluoride (NaF) induced oxidative stress and immunotoxicity in rats. Additionally, excessive fluoride ingestion induced the histological changes in rabbit spleens [32].

It has been reported that excessive fluoride reduced peripheral blood percentages of T-lymphocytes and B-lymphocytes and serum interleukin (IL)-2 and IL-6 concentrations in rabbits [33]. Our previous study also has demonstrated that high fluoride reduced the peripheral blood T-lymphocyte percentage and serum IL-2 in broiler chickens [34]. Our previous study found that fluoride impaired the splenic function *in vivo* and *in vitro* [35-37]. It has been proved that excessive sodium fluoride exposure suppresses splenic lymphocyte proliferation, which is represented by reducing populations and activation of splenic T and B lymphocytes. Alterations of cytokine protein expression and cell cycle arrest were the molecular basis of sodium fluoride (NaF)-suppressed splenic lymphocyte proliferation, while reduction of T and B lymphocytes were the cause of the decreased splenic immune function *in vitro* [37]. However, up to now, there were no systematic studies on high fluoride-induced blood cellular immunity and humoral immunity in mice. The current study investigated the effect of different concentrations of NaF treatment on blood T cell and B cell immune function. The blood T and B lymphocyte population, immunoglobulins and Th1/Th2/Th17 cytokines were observed in this study. We found that NaF induced both blood cellular and humoral immune function in mice, providing an excellent animal model for clinical studies on immunotoxicity related to fluorosis.

## RESULTS

### Changes of T-cell subsets and CD4^+^/CD8^+^ ratio in the peripheral blood

We detected the percentages of CD3^+^, CD4^+^, and CD8^+^ T-cell subsets by flow cytometry in the peripheral blood. The CD3^+^ T-cell is the mature T cells. CD4^+^ and CD8^+^ T-cell are T helper (Th) cells and T cytotoxic (Tc) cells.

**Figure 1:**
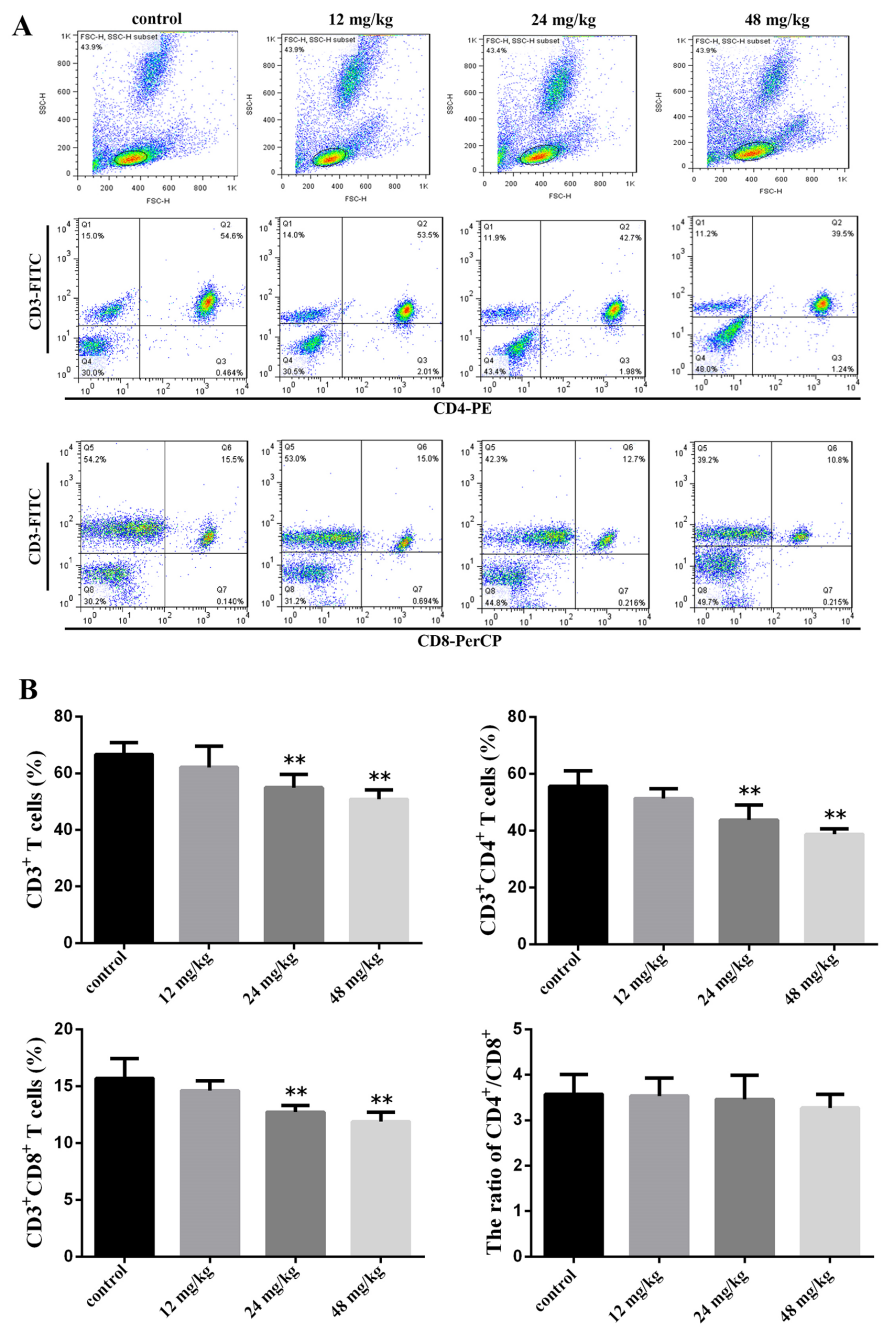
Changes of peripheral blood T-cell subsets and CD4+/CD8+ ratio at 21 days of age **(A)** Representative flow cytometric diagram of CD3^+^, CD4^+^ and CD8^+^ T-cell analysis. **(B)** Changes of the percentages of CD3^+^, CD4^+^ and CD8^+^ T-cell and CD4^+^/CD8^+^ ratio. Gate cells number 10^4^. Data are presented with the mean ± standard deviation (n=8). *P<0.05, compared with the control group; **P<0.01, compared with the control group.

At 21 days of experiment, the CD3^+^ T cells, CD3^+^CD4^+^ T cells and CD3^+^CD8^+^ T cells percentages were significantly reduced (*P*<0.01) in the 24 mg/kg and 48 mg/kg groups when compared with those in the control group. The ratio of CD4^+^/CD8^+^ T cells was no significant change between the control group and NaF-treated groups. The results were shown in Figure 1B.

**Figure 2:**
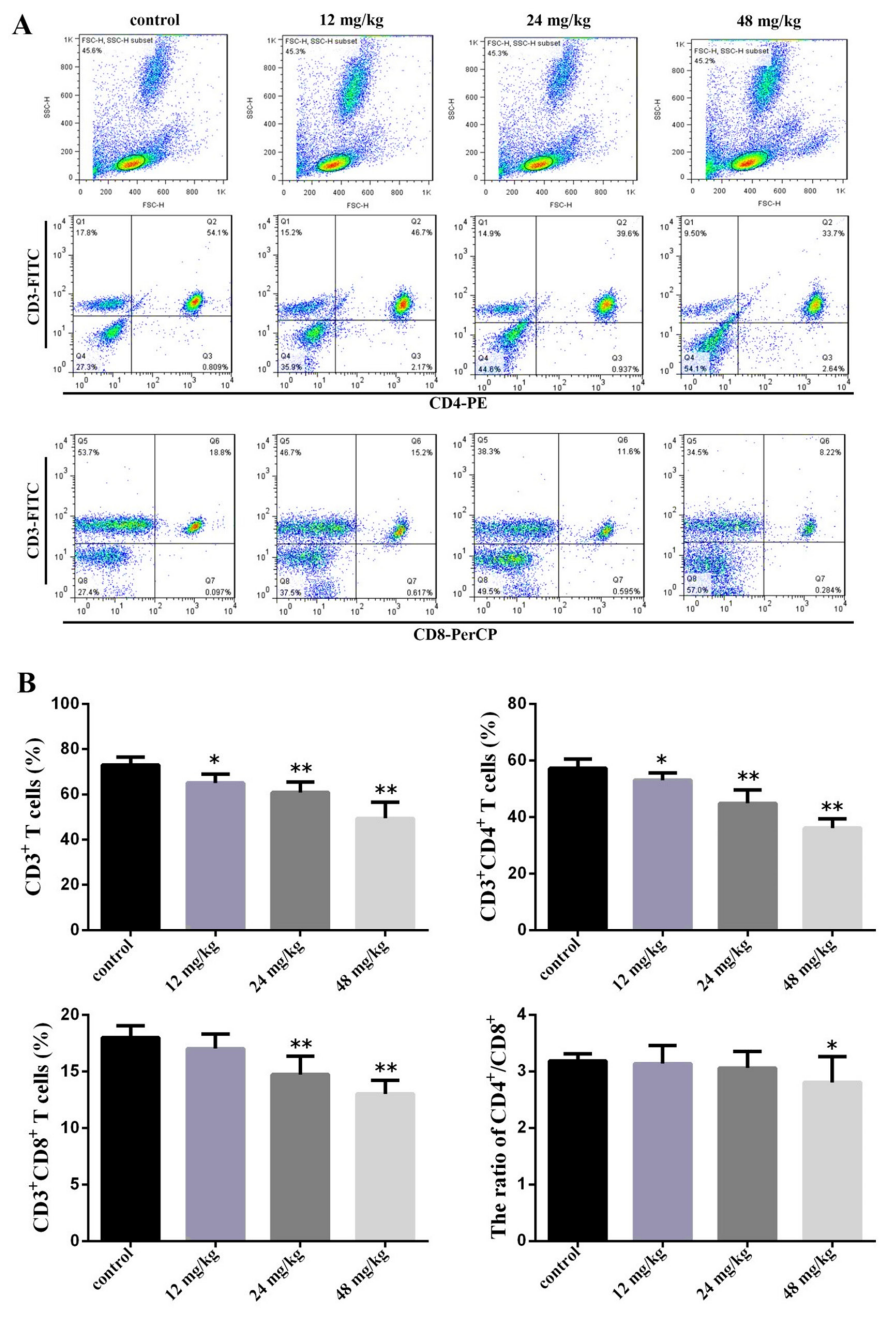
Changes of peripheral blood T-cell subsets and CD4+/CD8+ ratio at 42 days of age **(A)** Representative flow cytometric diagram of CD3^+^, CD4^+^ and CD8^+^ T-cell analysis. **(B)** Changes of the percentages of CD3^+^, CD4^+^ and CD8^+^ T-cell and CD4^+^/CD8^+^ ratio. Data are presented with the mean ± standard deviation (n=8). *P<0.05, compared with the control group; **P<0.01, compared with the control group.

As shown in Figure 2B at 42 days of experiment, the percentages of CD3^+^ T cells, CD3^+^CD4^+^ T cells were significantly lower (*P*<0.05 and *P*<0.01) in the three NaF-treated groups than those in the control group. The percentages of CD3^+^CD8^+^ T cells were significantly decreased (*P* <0.01) in the 24 mg/kg and 48 mg/kg groups when compared with those in the control group. The ratio of CD4+/CD8+ T cells was significantly decreased (*P*<0.01) in the 48 mg/L group when comparison with those in the control group. The absolute numbers of CD3^+^, CD4^+^, and CD8^+^ T-cell were showed in the Table 1.

**Table 1:** Changes of absolute numbers of peripheral blood T lymphocyte subsets

		21 days of age	42 days of age
CD3	Control	8704.35±544.92	9846.00±479.56
	12mg/kg	8106.23±983.50	8784.00±524.43*
	24mg/kg	7170.98±623.99**	8221.50±608.85**
	48mg/kg	6635.93±432.17**	6680.25±955.11**
CD3CD4	Control	7264.50±702.80	7722.00±441.76
	12mg/kg	6694.65±466.30	7159.50±347.10
	24mg/kg	5711.55±685.28**	6052.50±648.21**
	48mg/kg	5046.00±248.38**	4875.75±441.96**
CD3CD8	Control	2048.85±222.54	2427.75±141.70
	12mg/kg	1905.30±114.36	2297.25±172.76
	24mg/kg	1659.53±74.93**	1986.98±220.54**
	48mg/kg	1550.12±105.31**	1755.00±162.45**

Data are presented with the mean ± standard deviation (n=8). *P<0.05, compared with the control group; **P<0.01, compared with the control group.

### Changes of the serum Th1/Th2/Th17 cytokines expression

CD4^+^ T-cell can be classified into Th1 cells, which produce IL-2, interferon (IFN)-γ and tumor necrosis factor (TNF) involved in cellular immunity, and Th2 cells, which produce IL-4, IL-6 and IL-10 involved in humoral immunity, and Th17 cells, which produce the proinflammatory cytokine, e.g., IL-17, play important roles in the induction of inflammation.

**Figure 3:**
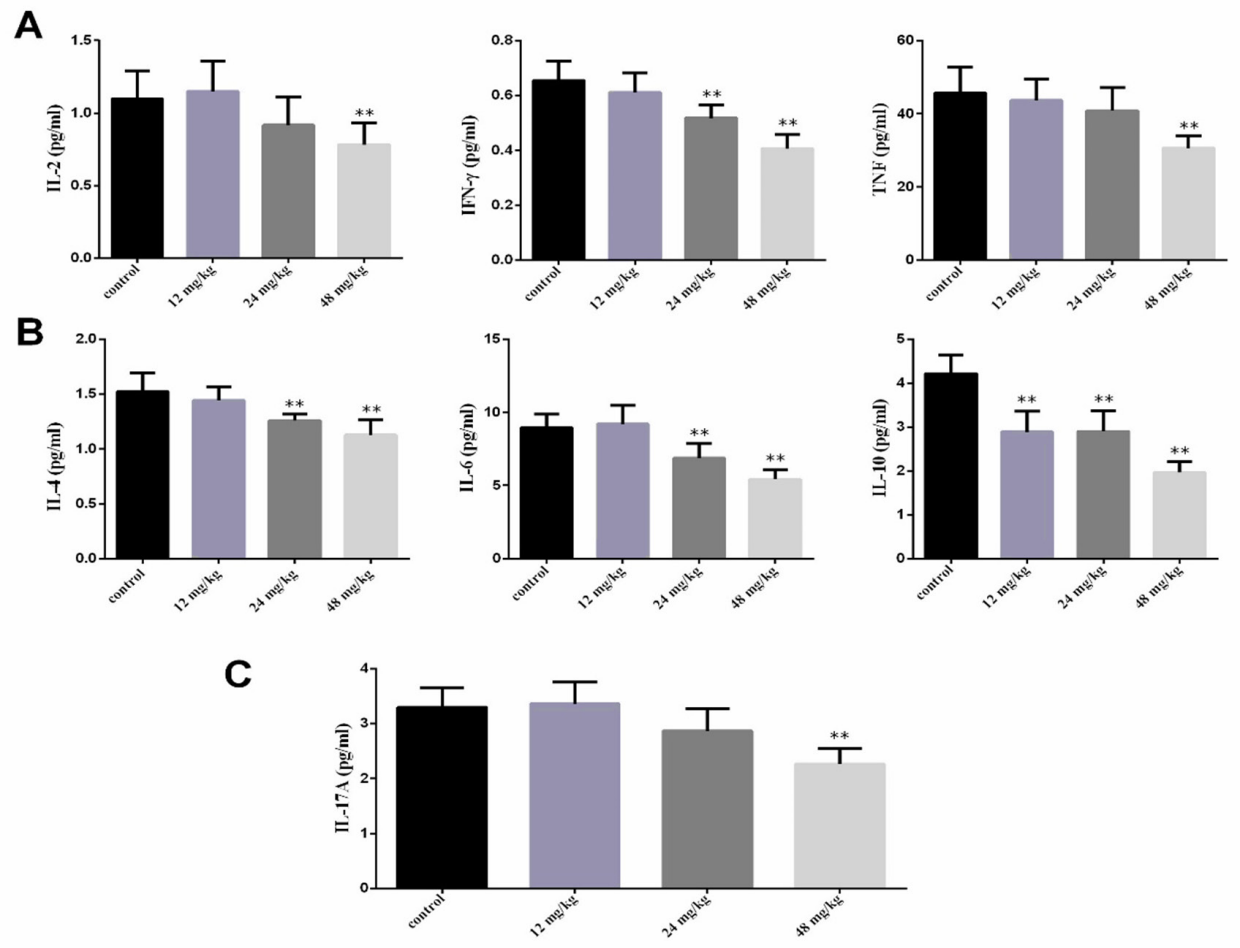
Changes of serum cytokines contents at 21 days of age **(A)** Changes of serum Th1 cytokines IL-2, IFN-γ and TNF contents. **(B)** Changes of serum Th2 cytokines IL-4, IL-6 and IL-10 contents. **(C)** Changes of serum Th17 cytokines IL-17A contents. Data are presented with the mean ± standard deviation (n=8). *P<0.05, compared with the control group; **P<0.01, compared with the control group.

The results showed that the IL-2, IFN-γ and TNF contents were significantly lower (*P*<0.01) in the 48 mg/kg group at 21 days of experiment and IFN-γ contents were significantly lower (*P*<0.01) in the 24 mg/kg group at 21 days of experiment than those in the control group. The IL-4, IL-6 and IL-10 contents were significantly decreased (*P*<0.01) in the 24 mg/kg and 48 mg/kg groups at 21 days of experiment and IL-10 contents were significantly reduced (*P*<0.01) in the 12 mg/kg group at 21 days of experiment. The IL-17A contents were significantly reduced (*P*<0.01) in the 48 mg/kg group at 21 days of experiment when compared with those in the control group, as shown in Figure 3.

**Figure 4:**
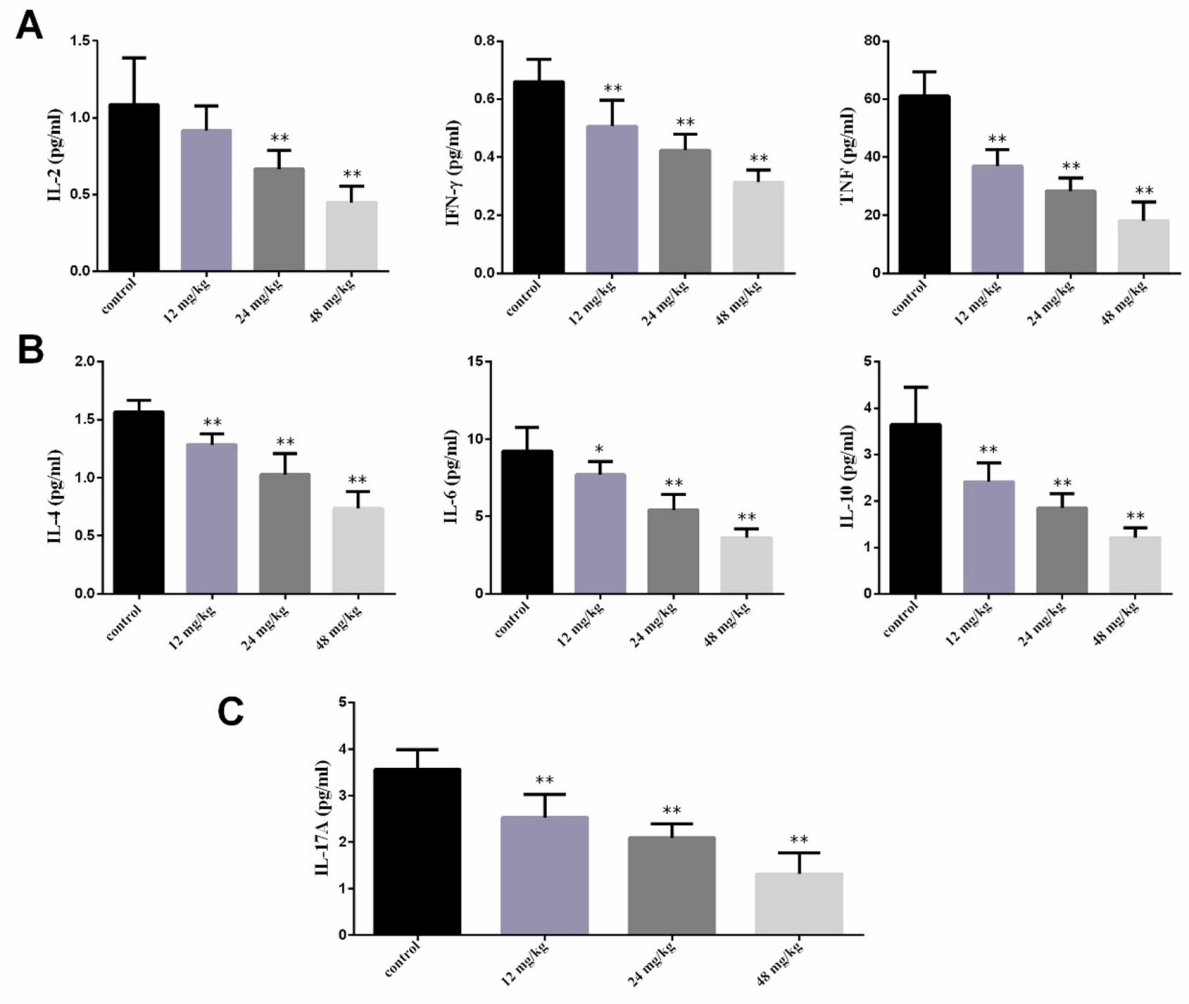
Changes of serum cytokines contents at 42 days of age **(A)** Changes of serum Th1 cytokines IL-2, IFN-γ and TNF contents. **(B)** Changes of serum Th2 cytokines IL-4, IL-6 and IL-10 contents. **(C)** Changes of serum Th17 cytokines IL-17A contents. Data are presented with the mean ± standard deviation (n=8). *P<0.05, compared with the control group; **P<0.01, compared with the control group.

In the Figure 4, the IL-2 contents were significantly lower (*P*<0.01) in the 24 mg/kg and 48 mg/kg groups at 42 days of experiment than those in the control group. The IFN-γ and TNF contents were significantly decreased (*P*<0.01) in the three NaF-treated groups at 42 days of experiment when compared with those in the control group. The IL-4, IL-6 and IL-10 contents were significantly reduced (*P*<0.01) in the three NaF-treated groups at 42 days of experiment in comparison with those in the control group. The IL-17A contents were significantly lower (*P*<0.01) in the three NaF-treated groups at 42 days of age than those in the control group.

### Changes of peripheral blood B-cell percentage and serum immunoglobulin A (IgA), immunoglobulin G (IgG), and immunoglobulin M (IgM) contents

We measured the B cell percentages and the antibodies (IgA, IgG and IgM) contents, which play important role in the humoral immunity.

**Figure 5:**
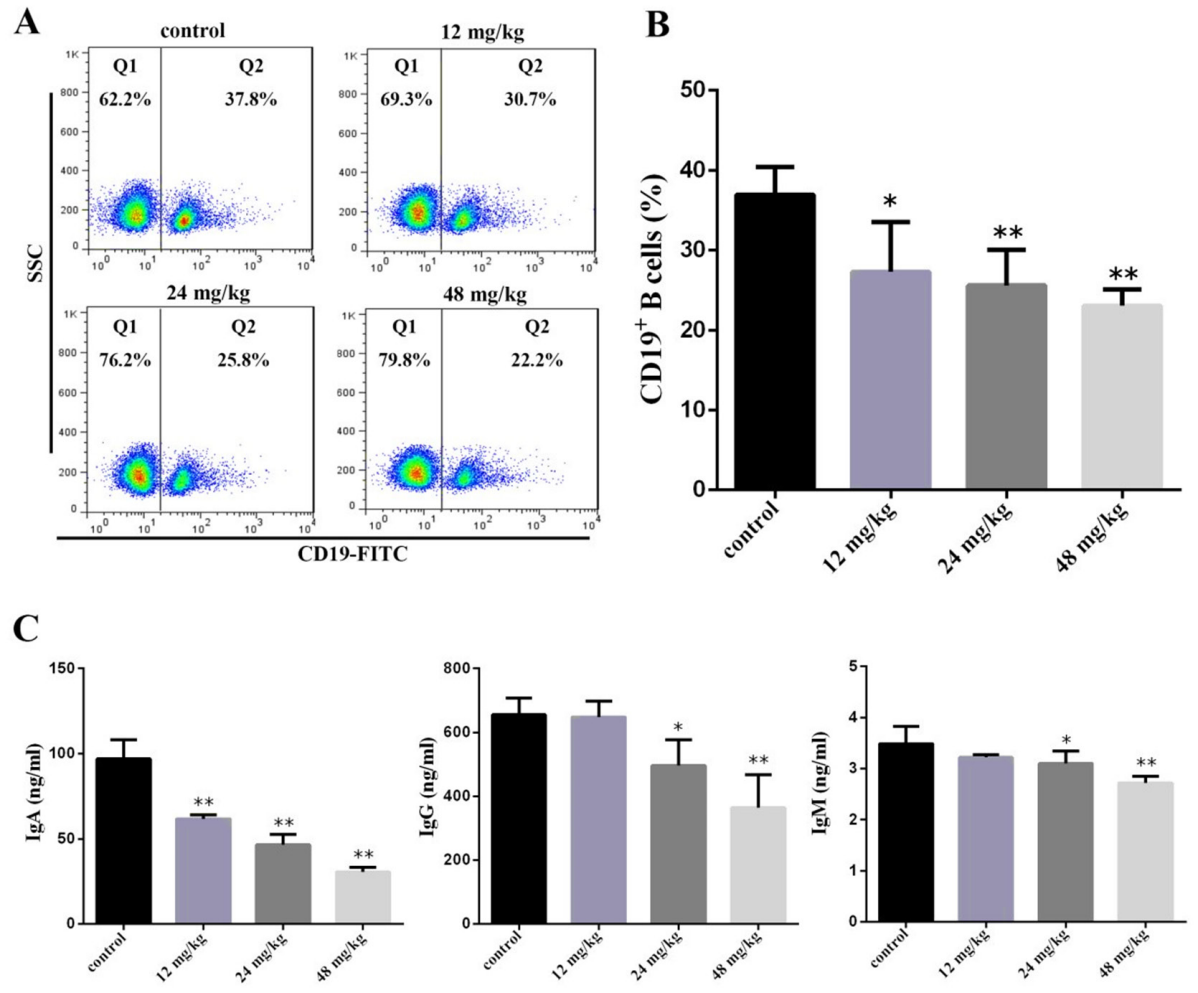
Changes of peripheral blood B-cell and serum IgA, IgG and IgM contents at 21 days of age **(A)** Representative flow cytometric diagram of CD19+ B-cell analysis. **(B)** Changes of the percentages of CD19 + B-cell. **(C)** Changes of serum IgA, IgG and IgM contents. Data are presented with the mean ± standard deviation (n=8). *P<0.05, compared with the control group; **P<0.01, compared with the control group.

At 21 days of experiment, the results in Figure 5B showed that the percentages of CD19+ B cells were significantly decreased (*P*<0.05 and *P*<0.01) in the three NaF-treated groups when compared with those in the control group. Figure 5C showed that the IgA contents were significantly lower (*P*<0.05 and *P*<0.01) in the three NaF-treated groups than those in the control group. The IgM and IgG contents were significantly reduced (*P*<0.05 and *P*<0.01) in the 24 mg/kg and 48 mg/kg groups when compared with those in the control group.

**Figure 6:**
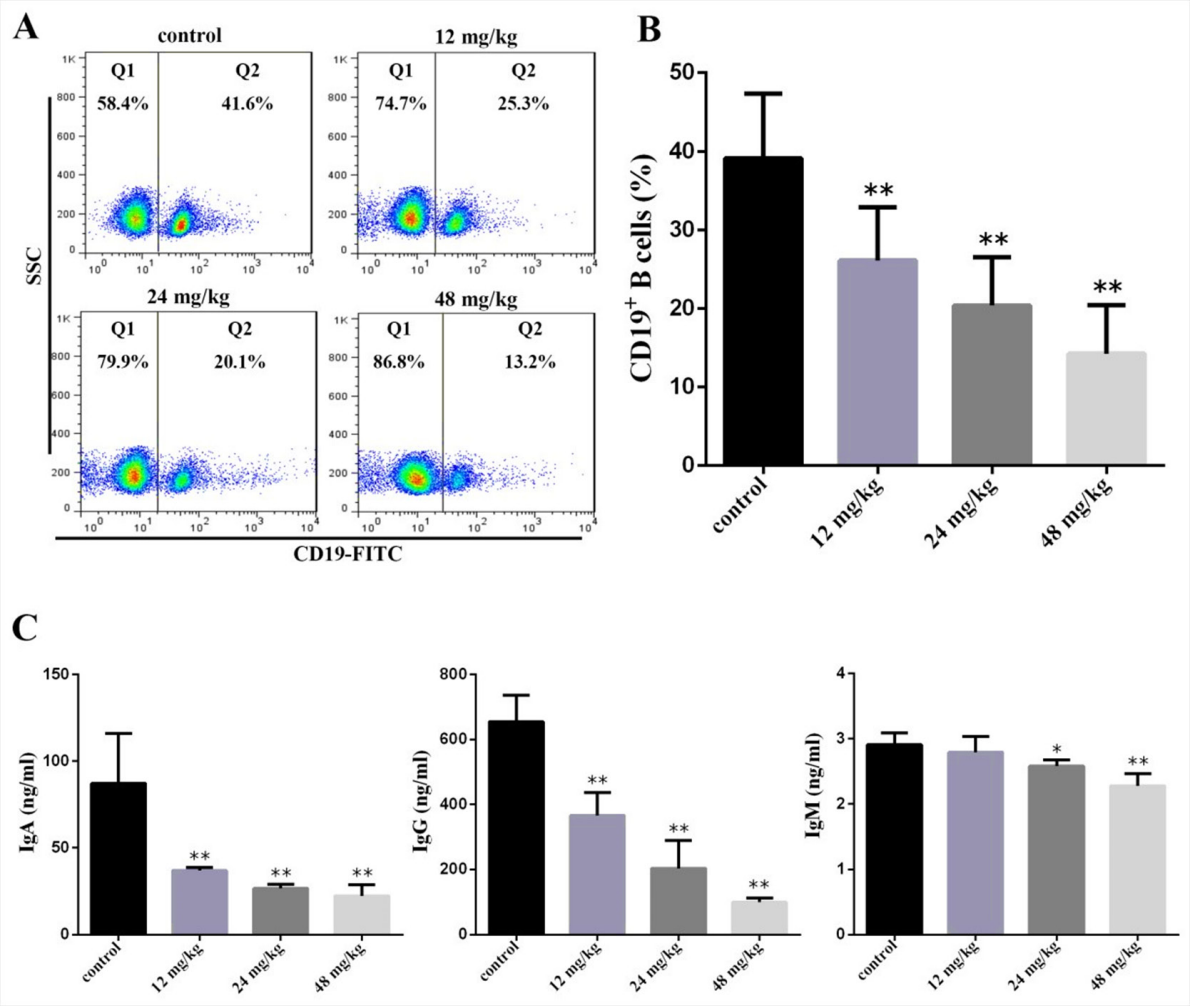
Changes of peripheral blood B-cell and serum IgA, IgG and IgM contents at 42 days of age **(A)** Representative flow cytometric diagram of CD19+ B-cell analysis. **(B)** Changes of the percentages of CD19 + B-cell. **(C)** Changes of serum IgA, IgG and IgM contents. Data are presented with the mean ± standard deviation (n=8). *P<0.05, compared with the control group; **P<0.01, compared with the control group.

At 42 days of experiment, the percentages of CD19+ B cells were significantly decreased (*P*<0.05 and *P*<0.01) in the three NaF-treated groups when compared with those in the control group. The IgA and IgM contents were significantly lower (*P*<0.01) in the three NaF-treated groups than those in the control group. The IgG contents were significantly decreased (*P*<0.05 and *P*<0.01) in the 24 mg/kg and 48 mg/kg group in comparison with those in the control group, as shown in Figure 6B and 6C.

Also, the absolute numbers of CD19+ B cells were showed in the Table 2.

**Table 2:** Changes of absolute numbers of peripheral blood CD19+ B lymphocytes

		21 days of age	42 days of age
CD19	Control group	1552.32±144.14	1642.90±344.39
	12mg/kg	1146.60±260.82**	1097.60±282.15**
	24mg/kg	1074.36±187.70**	855.40±259.30**
	48mg/kg	968.52±85.69**	599.13±259.85**

Data are presented with the mean ± standard deviation (n=8). *P<0.05, compared with the control group; **P<0.01, compared with the control group.

## DISCUSSION

Changes in hematological immune parameters can directly represent the immune function of the body [38]. Immunity is divided into the innate and the adaptive responses. The adaptive immune system includes T and B lymphocytes, specific antibodies, cytokines and chemokines [39]. In this study, the results showed that NaF reduced the blood cellular and humoral immune function in mice.

In cellular immunity, our results showed that NaF induced a significant decrease in the percentages of CD3^+^, CD3^+^CD4^+^, CD3^+^CD8^+^ T lymphocytes and CD4^+^/CD8^+^ ratio in the peripheral blood. Our previous studies demonstrated that high fluoride intake decreased the percent of peripheral blood CD4^+^ and CD8^+^ percentage and serum IL-2 levels in the chickens [34]. It should also be noted that T lymphocytes play a role in both cellular immunity and antibody generation. In general, T lymphocytes are divided into two subsets Th1 and Th2 cells [40]. Th1 cells contribute to cytotoxic T cell differentiation and mediate cellular immune responses. Th1 cytokines include IL-2, IFN-γ, and TNF. Th2 cells secrete IL-4, IL-5, IL-6, IL-10 and IL-13. Th2 cells contribute to promote B cell proliferation and differentiation and up-regulation of antibody production [41]. Th2 cells play an important role in humoral immune respons [40]. In this study, NaF decreased serum Th1 cytokines (IL-2, IFN-γ, TNF) and Th2 cytokines (IL-4, IL-6, IL-10), which were consistent with the changes of CD3^+^CD4^+^. Recently, Th17 cells are discovered and found mainly to produce IL-17. Th17 cells can clear pathogens that are not adequately removed by Th1 or Th2 cells [42]. We also measured the Th17 and found that IL-17A content was decreased after exposure to NaF. In our previous study, we found that high fluoride levels can reduce IL-2, IL-4, IL-6, TNF-α and IFN-γ in the cecal tonsil, and intestines of broiler chickens [14], and that NaF can reduce the percentages of CD3^+^, CD3^+^CD4^+^, CD3^+^CD8^+^ T lymphocytes and CD19^+^ B lymphocytes and of IL-2, TNF-α, IFN-γ, TGF-β expression levels in cultured splenic lymphocytes of mice [36]. Our findings are consistent with the report of Yin et al. [43] that high fluoride decreased the percentages of thymic CD4^+^, CD8^+^ T cells and IL-2, IL-10 mRNA expression in mice. Also, Fentue et al. [19] reported that NaF decreased IL-10 mRNA expression level in murine macrophages. The decrease in T lymphocytes and Th1/Th2/Th17 cytokines indicate that excess intake of NaF can suppress the cellular immunity in mice and inhibit IL-4, IL-6, IL-10. Meanwhile, our results also showed down-regulation of CD19^+^ B lymphocytes in the peripheral blood and serum IgA, IgG and IgM. Our previous studies have found that high fluorine levels decrease the IgA, IgG and IgM in the cecal tonsil, intestines of broiler chickens [15, 30]. Our results are in line with the reports of Jain et al. [44] who demonstrated that orally NaF inhibited antibody formation and decreased the proliferation of lymphocytes in rabbits. It is well known that B cells secrete IgA, IgG and IgM, which are important antibodies involved in humoral immunity [45, 46]. B lymphocyte inhibition is the main reason for downregulation of IgA, IgG and IgM. Additinally, reduction of the Th2 cytokines can also inhibit the secretion of IgA, IgG and IgM. The decrease in B lymphocytes and IgA, IgG and IgM indicate that excessive NaF intake can suppress the humoral immunity in mice.

In this study, the decrease in the percent of CD3^+^, CD3^+^CD4^+^, CD3^+^CD8^+^ T lymphocytes and CD19^+^ B lymphocytes in the peripheral blood may be due to NaF-induced T and B lymphocyte apoptosis or suppressed lymphocyte proliferation. Our previous study demonstrated that NaF could induce splenic T and B lymphocytes apoptosis and inhibition of proliferation *in vitro* and *vivo* [35-37]. NaF-induced apoptosis in splenic lymphocytes could be mediated by mitochondrial and death receptor pathways [35]. Some studies have demonstrated that fluoride can induce immune cell apoptosis [47, 48]. NaF can induce chromosome damage and apoptosis in human lymphocyte cells *in vitro* [49, 50], and apoptosis in human bone marrow and cord blood CD 34 positive cells [51]. Song et al. [52] reported that high dosage of NaF can inhibit cell proliferation by stress-induced apoptosis in Leydig cells. Fluoride can cause cell cycle aberration, apoptosis, and DNA damage in cultures of TM3 mouse Leydig cells [53].

## CONCLUSION

The above results show that NaF negatively affects blood cellular and humoral immune function in mice, which is shown by the reduction of T and B lymphocyte populations, immunoglobulins, and Th1/Th2/Th17 cytokines. This study provides an excellent animal model for clinical studies on immunotoxicity-related fluorosis.

## MATERIALS AND METHODS

### Animals and treatment

240 healthy four week-old male and female ICR mice (Experimental Animal Corporation of DOSSY at Chengdu, China) were used in this experiment. Food and water was provided *ad libitum*. The mice were divided into 4 groups (N=60). After one week of acclimatization, the control group was given an intragastric administration of distilled water at the same time as other groups. The experimental groups were given an intragastric administration of 12, 24, and 48 mg/kg NaF (Chengdu Kelong Chemical Co., Ltd., Chengdu, China), respectively, NaF was diluted by distilled water. The gavage doses of four groups were 1 mg/100g animal weight once daily for the last 42 days.

Our experiments involving the use of mice and all experimental procedures were approved by the Animal Care and Use Committee, Sichuan Agricultural University.

### Observation of T-cell percentages in the peripheral blood

At 21 and 42 days, the blood samples were taken from retro-ocular artery. The peripheral blood of eight mice in each group were taken to measure the percentages of CD3^+^, CD3^+^CD4^+^, CD3^+^CD8^+^ T lymphocyte by flow cytometry.

100 μL anti-clotting peripheral blood was put in a test tube. And then added 10 μL hamster anti-mouse CD3e-FITC (BD, Cat No: 553062), rat anti-mouse CD4-PE (BD, Cat No: 557308) and rat anti-mouse CD8a-PerCP (BD, Cat No: 553036) to the test tube for 30 min at RT, and then 1 mL lysing solution (BD, Cat No: 349202) was added for 10 min at room temperature. Subsequently, 1 mL PBS was added and centrifuged at 200×g for 5 min. The supernatant was discarded. 2 mL PBS was added and centrifuged at 200×g for 5 min and the supernatant was discarded. The cells were resuspended in 0.5 mL PBS and determined by BD FACS Calibur flow cytomyter.

### Observation of serum Th1/Th2/Th17 cytokines

At 21 and 42 days, the mice blood samples were taken from retro-ocular artery. The serum was obtained by blood centrifugation (3,500 × g, 15 min).

The concentrations of serum cytokines were measured using a BD Cytometric Bead Array (CBA) Mouse Th1/Th2/Th17 Cytokine Kit (BD, Cat No: 560485) according to the manufacturer’s protocol. Cytokine Limit of detection (pg/mL): IL-2, 0.1; IL-4, 0.03; IL-6, 1.4; IFN-g, 0.5; TNF, 0.9; IL-17A, 0.8; IL-10, 16.8. 50 μL serum or standards were incubated with 50 μL of capture beads for 1h at room temperature, and then mixed with 50 μL of phycoerythrin (PE)-conjugated IL-2, IL-4, IL-6, IL-10, IL-17A, TNF, and IFN-γ antibodies and incubated for 2 h at room temperature to form a sandwich complex. Following incubation, 1 mL washing buffer was added to each tube, and centrifuged at 200×g for 5 min. The supernatant was discarded. 500 μL washing buffer was added to each tube and the mean fluorescence intensity was detected using BD FACS Calibur flow cytomyter. Data were analyzed using FACP array software. The version of FCAP is BD FACP array software v3.0.

### Observation of B-cell in the peripheral blood

At 21 and 42 days, the peripheral blood of eight mice in each group was taken to determine the percentages of CD 19+ B lymphocyte by flow cytometry.

Anti-clotting peripheral blood (100 μL) was put in a test tube. The cells were stained with 10 μL rat anti-mouse CD19-FITC (BD, Cat No: 553785) for 30 min at RT, and then 1 mL lysing solution was added for 10 min at RT. Subsequently, 1 mL PBS was added and centrifuged at 200×g for 5 min. The supernatant was discarded. 2 mL PBS was added and centrifuged at 200×g for 5 min and the supernatant was discarded. The cells were resuspended in 0.5 mL PBS and determined by BD FACS Calibur flow cytomyter.

### Observation of serum IgA, IgG, and IgM

At 21 and 42 days, the mice blood samples were taken from retro-ocular artery. The serum was obtained by blood centrifugation (3,500 × g, 15 min).

The serum IgA, IgG, and IgM contents were determined by using ELISA as described by Gaca et al. [54]. Those serum immunoglobulins were quantified by using the IgA kit (Nanjing Jiancheng Bioengineering Institute, Cat No: H108), IgG kit (Nanjing Jiancheng Bioengineering Institute, Cat No: H106), IgM kit (Nanjing Jiancheng Bioengineering Institute, Cat No: H109).

### Statistical analysis

The experimental data are expressed as the mean ± standard deviation. One-way analysis of variance (ANOVA) procedure in SPSS 17.0 software was used to assess statistical significances between NaF-treated group and control group. A value of *P*<0.05 was considered significant, and *P*<0.01 was markedly significant.

## References

[1] Hagmann WK (2008). The many roles for fluorine in medicinal chemistry. J Med Chem.

[2] Strunecka A, Patocka J, Connett P (2004). Fluorine in medicine. J Appl Biomed.

[3] Malayeri BE, Noori M, Jafari M (2012). Using the pollen viability and morphology for fluoride pollution biomonitoring. Biol Trace Elem Res.

[4] Li P, Xue Y, Zhang W, Teng F, Sun Y, Qu T, Chen X, Cheng X, Song B, Luo W, Yu Q (2013). Sodium fluoride induces apoptosis in odontoblasts via a JNK-dependent mechanism. Toxicology.

[5] Cao A, Guo M, Yan D, Mao L, Wang Q, Li Y, Duan X, Wang P (2014). Evaluation of sulfuryl fluoride as a soil fumigant in China. Pest Manag Sci.

[6] ATSDR (Agency forToxicSubstances and Disease Registry) (September 2003). Toxicological profile for fluorides, hydrogen fluoride, and fluorine. U.S. Department of Health and Human Services.

[7] Del Piero S (2013). Fluoride toxicity. Environ Toxicol Chem.

[8] Adamek E, Pawłowska-Góral K, Bober K (2005). [*in vitro* and *in vivo* effects of fluoride ions on enzyme activity]. [Article in English, Polish]. Ann Acad Med Stetin.

[9] Barbier O, Arreola-Mendoza L, Del Razo LM (2010). Molecular mechanisms of fluoride toxicity. Chem Biol Interact.

[10] Gong T, Chen T, Bai C, Peng X, Cui H (2009). Effect of dietary high fluorine on the cell cycle and apoptosis of liver in chickens. Chin J Anim Vet Sci.

[11] Gong T, Bai C, Chen T, Peng X, Cui H (2009). Effect of high fluorine on the antioxidant function and ultrastructure of liver in chickens. Chin J Anim Vet Sci.

[12] Bai C, Chen T, Cui Y, Gong T, Peng X, Cui HM (2010). Effect of high fluorine on the cell cycle and apoptosis of renal cells in chickens. Biol Trace Elem Res.

[13] Luo Q, Cui H, Peng X, Fang J, Zuo Z, Deng J, Liu J, Deng Y (2016). Dietary high fluorine alters intestinal microbiota in broiler chickens. Biol Trace Elem Res.

[14] Luo Q, Cui H, Peng X, Fang J, Zuo Z, Liu J, Wu B, Deng Y (2013). The association between cytokines and intestinal mucosal immunity among broilers fed on diets supplemented with fluorine. Biol Trace Elem Res.

[15] Luo Q, Cui H, Peng X, Fang J, Zuo Z, Deng J, Liu J, Deng Y (2013). Intestinal IgA (+) cell numbers as well as IgA, IgG, and IgM contents correlate with mucosal humoral immunity of broilers during supplementation with high fluorine in the diets. Biol Trace Elem Res.

[16] Luo Q, Cui H, Peng X, Fang J, Zuo Z, Deng J, Liu J, Deng Y (2013). Suppressive effects of dietary high fluorine on the intestinal development in broilers. Biol Trace Elem Res.

[17] Gandhi D, Naoghare PK, Bafana A, Kannan K, Sivanesan S (2017). Fluoride-induced oxidative and inflammatory stress in osteosarcoma cells: does it affect bone development pathway?. Biol Trace Elem Res.

[18] Yang Y, Huang H, Ba Y, Cheng XM, Cui LX (2015). Effect of oxidative stress on fluoride-induced apoptosis in primary cultured Sertoli cells of rats. Int J Environ Health Res.

[19] De la Fuente B (2016). Vazquez M, Rocha RA, Devesa V, Velez D. Effects of sodium fluoride on immune response in murine macrophages. Toxicol *In Vitro*.

[20] Das SS, Maiti R, Ghosh D (2006). Fluoride-induced immunotoxicity in adult male albino rat: a correlative approach to oxidative stress. J Immunotoxicol.

[21] Sutton PR (1987). Does fluoride ingestion affect developing immune system cells?. Med Hypotheses.

[22] Gibson SL (1998). Effects of fluoride on immune system function. Community Dental Health.

[23] Hernandez-Castro B, Vigna-Perez M, Doniz-Padilla L, Ortiz-Perez MD, Jimenez-Capdeville E, Gonzalez-Amaro R, Baranda L (2011). Effect of fluoride exposure on different immune parameters in humans. Immunopharmacol Immunotoxicol.

[24] Chen T, Cui H, Cui Y, Bai C, Gong T, Peng X (2011). Cell-cycle blockage associated with increased apoptotic cells in the thymus of chickens fed on diets high in fluorine. Human Exp Toxicol.

[25] Chen T, Cui H, Cui Y, Bai C, Gong T (2011). Decreased antioxidase activities and oxidative stress in the spleen of chickens fed on high-fluorine diets. Human Exp Toxicol.

[26] Chen T, Cui Y, Bai C, Gong T, Peng X, Cui H (2009). Increased apoptotic lymphocyte population in the spleen of young chickens fed diets high in fluorine. Fluoride.

[27] Chen T, Cui Y, Gong T, Bai C, Peng X, Cui H (2009). Inhibition of splenocyte proliferation and spleen growth in young chickens fed high fluoride diets. Fluoride.

[28] Chen T, Gong T, Bai C, Peng X, Cui H (2009). Effect of dietary high fluorine on the morphologic structure, cell cycle and apoptosis of bursa of fabricius in broilers. Chin J Anim Vet Sci.

[29] Liu J, Cui H, Peng X, Fang J, Zuo Z, Wang H, Wu B, Deng Y, Wang K (2013). Dietary high fluorine induces apoptosis and alters Bcl-2, Bax, and caspase-3 protein expression in the cecal tonsil lymphocytes of broilers. Biol Trace Elem Res.

[30] Liu J, Cui H, Peng X, Fang J, Zuo Z, Deng J, Wang H, Wu B, Deng Y, Wang K (2013). Decreased IgA+ B cells population and IgA, IgG, IgM contents of the cecal tonsil induced by dietary high fluorine in broilers. Int J Environ Res Public Health.

[31] Wang H, Zhou B, Cao J, Gu X, Cao C, Wang J (2009). Effects of dietary protein and calcium on thymus apoptosis induced by fluoride in female rats (Wistar rats). Environ Toxicol.

[32] Zhou B, Wang H, Wang J, Zhang J, Yan X, Wang J (2007). Effects of malnutrition and supplemented nutrition on nonspecific immune function changes induced by fluoride in rabbits. Fluoride.

[33] Zhou B, Wang H, Wang J, Zhang J, Yan X, Wang J (2009). Effects of malnutrition and supplemented nutrition on specific immune parameter changes induced by fluoride in rabbits. Fluoride.

[34] Chen T, Cui Y, Bai C, Gong T, Peng X, Cui H (2009). Decreased percentages of the peripheral blood T-cell subsets and the serum IL-2 contents in chickens fed on diets excess in fluorine. Biol Trace Elem Res.

[35] Deng H, Kuang P, Cui H, Chen L, Fang J, Zuo Z, Deng J, Wang X, Zhao L (2016). Sodium fluoride induces apoptosis in cultured splenic lymphocytes from mice. Oncotarget.

[36] Kuang P, Deng H, Cui H, Chen L, Guo H, Fang J, Zuo Z, Deng J, Wang X, Zhao L (2016). Suppressive effects of sodium fluoride on cultured splenic lymphocyte proliferation in mice. Oncotarget.

[37] Kuang P, Deng H, Cui H, Chen L, Fang J, Zuo Z, Deng J, Wang X, Zhao L (2017). Sodium fluoride (NaF) causes toxic effects on splenic development in mice. Oncotarget.

[38] Toghyani M, Tohidi M, Gheisari AA, Tabeidian SA (2010). Performance, immunity, serum biochemical and hematological parameters in broiler chicks fed dietary thyme as alternative for an antibiotic growth promoter. Afr J Biotechnol.

[39] Iwasaki A, Medzhitov R (2015). Control of adaptive immunity by the innate immune system. Nat Immunol.

[40] Parkin J, Cohen B (2001). An overview of the immune system. Lancet.

[41] Niemeyer M, Darmoise A, Mollenkopf HJ, Hahnke K, Hurwitz R, Besra GS, Schaible UE, Kaufmann SH (2008). Natural killer T-cell characterization through gene expression profiling: an account of versatility bridging T helper type 1 (Th1), Th2 and Th17 immune responses. Immunology.

[42] Korn T, Bettelli E, Oukka M, Kuchroo VK (2009). IL-17 and Th17 cells. Annu Rev Immunol.

[43] Yin S, Wu H, Song C, Chen X, Zhang Y (2016). Modulation and the underlying mechanism of T cells in thymus of mice by oral administration of sodium fluoride. Biol Trace Elem Res.

[44] Jain SK, Susheela AK (1987). Effect of sodium fluoride on antibody formation in rabbits. Environ Res.

[45] Bruno PP, Carpino F, Carpino G, Zicari A (2007). An overview on immune system and migraine. Eur Rev Med Pharmacol Sci.

[46] Mauri C, Bosma A (2012). Immune regulatory function of B cells. Annu Rev Immunol.

[47] Singh R, Banerjee C, Ray A, Rajamani P, Mazumder S (2016). Fluoride-induced headkidney macrophage cell apoptosis involves activation of the CaMKII g-ERK 1/2-caspase-8 axis: the role of superoxide in initiating the apoptotic cascade. Toxicol Res.

[48] Li Y, Liang C, Katz B, Brizendine E, Stookey G (1995). Long-term exposure to fluoride in drinking water and sister chromatid exchange frequency in human blood lymphocytes. J Dental Res.

[49] Jothiramajayam M, Sinha S, Ghosh M, Nag A, Jana A, Mukherjee A (2014). Sodium fluoride promotes apoptosis by generation of reactive oxygen species in human lymphocytes. J Toxicol Environ Health, Part A.

[50] Albanese R (1987). Sodium fluoride and chromosome damage (*in vitro* human lymphocyte and *in vivo* micronucleus assays). Mutagenesis.

[51] Machalinska A, Machoy-Mokrzynska A, Marlicz W, Stecewicz I, Machalinski B (2001). NaF-induced apoptosis in human bone marrow and cord blood CD34 positive cells. Fluoride.

[52] Song G, Wang RL, Chen ZY, Zhang B, Wang HL, Liu ML, Gao JP, Yan XY (2014). Toxic effects of sodium fluoride on cell proliferation and apoptosis of Leydig cells from young mice. J Physiol Biochem.

[53] Song C, Cao X, Yang Z, Guo S, Shang Z (2013). Fluoride-induced cell cycle arrest, apoptosis, and DNA damage in TM3 mouse Leydig cells. Fluoride.

[54] Gaca MD, Pickering JA, Arthur MJ, Benyon RC (1999). Human and rat hepatic stellate cells produce stem cell factor: a possible mechanism for mast cell recruitment in liver fibrosis. J Hepatol.

